# Sleepless nights and social plights: medial septum GABAergic hyperactivity in a neuroligin 3–deficient autism model

**DOI:** 10.1172/JCI184795

**Published:** 2024-10-01

**Authors:** Claire E. Cho, Dahee Jung, Reesha R. Patel

**Affiliations:** Department of Psychiatry and Behavioral Sciences, Feinberg School of Medicine, Northwestern University, Chicago, Illinois, USA.

## Abstract

Social deficits represent a core symptom domain of autism spectrum disorder (ASD), which is often comorbid with sleep disturbances. In this issue of the *JCI*, Sun et al. explored a medial septum (MS) circuit linking these behaviors in a neuroligin 3 conditional knockout model of autism. They identified GABAergic neuron hyperactivity following neuroligin 3 deletion in the MS. This hyperactivity resulted in the inhibition of the downstream preoptic area (POA) and hippocampal CA2 region, resulting in sleep loss and social memory deficits, respectively. Inactivating the hyperactive MS GABA neurons or activating the POA or CA2 rescued the behavioral deficits. Together, these findings deepen our understanding of neural circuits underlying social and sleep deficits in ASD.

## Sociability and sleep in autism spectrum disorder

Autism spectrum disorder (ASD) is a complex neurodevelopmental disorder characterized by social interaction difficulties, repetitive behaviors, and restricted interests (1). Affecting approximately 1 in 36 children in the United States, ASD is four times more common in males than females (2). The genetic contributions to ASD are substantial; the Simons Foundation Autism Research Initiative gene database cites over 1000 candidate genes, underscoring the importance of ongoing genetic research to fully understand this disorder.

Many of these candidate genes are crucial for synaptic development, signaling, and plasticity (3), suggesting synaptic dysfunction in ASD. Notably, one of these synaptic genes encodes neuroligin 3 (*NLG3*), a cell-adhesion molecule required to organize synapses and enable efficient neurotransmission (3, 4). Both gain-of-function mutations and deletions in *NLGN3* are observed in ASD (5). Studies show that *Nlg3*-deficient mice have social interaction impairments (6), social memory deficits (7), and enhanced formation of repetitive routines (8), paralleling ASD-like behaviors.

In addition to social difficulties, 50%–80% of individuals with ASD suffer from sleep disorders (1). The relationship between social interaction and sleep is bidirectional; sleep deprivation can cause social withdrawal (9), and social isolation can negatively impact sleep (10). Studies revealed that *Nlg3*-knockout rats have reduced social interactions and sleep quality (11). Despite these observations, the neural circuitry linking sleep and social behavior in ASD remain unclear. Understanding these connections is crucial for developing targeted ASD interventions addressing both behavioral deficits.

One brain region that may play a key role in ASD is the medial septum (MS). Previous research has implicated the MS in regulating the sleep-wake cycle (12, 13) and modulating social behaviors (14, 15). However, whether the same MS neurons are involved in both behaviors in an *Nlg3*-knockout model of autism remains unknown. In this issue of the *JCI*, Sun and colleagues investigated this question, exploring whether conditional knockout (cKO) of *Nlg*3 in the MS might underlie the sleep and social disturbances seen in ASD (16).

## MS^GABA^ hyperactivity causes social avoidance and wakefulness

The authors explored the role of MS NLG3 in social behavior and sleep patterns by first conditionally knocking out *Nlg3* (*Nlg3*-cKO) in the MS of mice. Using the social novelty test, they found that *Nlg3*-cKO mice displayed normal sociability in comparison with controls, but they showed diminished interest in an unfamiliar mouse, indicative of social memory impairments. Additionally, *Nlg3*-cKO mice exhibited reduced non–rapid eye movement (NREM) and rapid eye movement (REM) sleep, accompanied by increased wakefulness.

To identify neurons active during social interactions and wakefulness, the authors next labeled the MS for *c-Fos*, a neural activity marker, and *Vgat*, a marker for the inhibitory neurotransmitter GABA. *Nlg3*-cKO mice showed higher levels of *c-Fos*–expressing neurons, with most coexpressing *Vgat*, indicating hyperactivity of the MS GABAergic neuron population (MS^GABA^).

Having identified this neural population, the authors used optrode recordings to monitor MS^GABA^ activity during social and sleep-wake behaviors. MS^GABA^ activity decreased when *Nlg3*-cKO mice approached an unfamiliar mouse, but increased when avoiding a familiar one. Control mice lacked encoding of approach or avoidance, suggesting that this response was specific to *Nlg3*-cKO mice. Furthermore, *Nlg3*-cKO mice showed greater MS^GABA^ baseline activity than controls during wake, NREM, and REM sleep, with increased firing during transitions from sleep to wake. To investigate the causal relationship between MS^GABA^ activity and altered behavior, the authors optogenetically activated or inactivated MS^GABA^ neurons. MS^GABA^ neuron activation in wild-type mice decreased interactions with an unfamiliar mouse, reduced NREM and REM sleep, and increased wakefulness. Conversely, MS^GABA^ neuron inactivation in *Nlg3*-cKO mice increased social novelty, sleep, and decreased wakefulness.

The bidirectional relationship between social interactions and sleep was further dissected by testing the effects of sleep deprivation and social isolation in wild-type mice. After six hours of sleep deprivation, mice showed no social novelty preference, decreased *Nlg3* mRNA levels, and increased MS^GABA^ activation, consistent with previous results. Similarly, four weeks of social isolation increased wakefulness, decreased *Nlg3* mRNA levels, and increased MS^GABA^ activation. These results reveal a dual, causal relationship between MS^GABA^ hyperactivity and social and sleep deficits.

## Distinct MS^GABA^ projections mediate social avoidance and wakefulness

The authors next sought to understand how MS^GABA^ circuitry affects social and sleep behaviors. One hypothesis is that distinct MS^GABA^ populations project to separate downstream regions, each regulating specific behaviors. Alternatively, the same MS^GABA^ neurons might relay identical information to various downstream targets through multiple collaterals. Unraveling these circuit motifs is essential for understanding how the MS orchestrates diverse behaviors.

To identify these MS^GABA^ projections, the researchers used viral anterograde tracing in *Nlg3*-cKO mice. They found MS projections to various brain regions, including the preoptic area (POA), which contains sleep-promoting neurons, and the hippocampal CA2 region, which is implicated in social memory. Viral retrograde tracing labeled specific MS neurons that projected to the POA and CA2 region. Interestingly, about 30% of these neurons overlapped, suggesting that some of these MS neurons indeed collateralize and possibly mediate the tight relationship between sleep and social memory.

**Figure 1:**
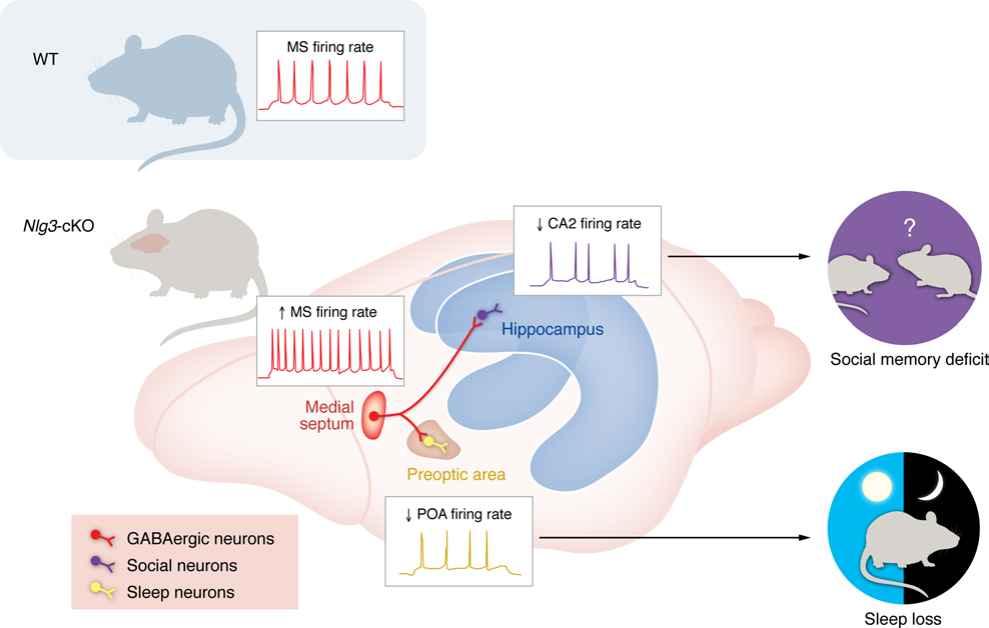
MS^GABA^ neurons regulate social memory and sleep in *Nlg3*-cKO mice. *Nlg3* knockout induces hyperactivity of GABA neurons in the MS. Hyperactivated MS^GABA^ neurons impair social memory by reducing CA2 activity and induces sleep loss by decreasing POA activity.

To establish a functional role for the POA and CA2, the authors optogenetically inhibited POA and CA2 neurons innervated by MS^GABA^ terminals in wild-type mice. CA2 inhibition impaired social memory without affecting the sleep-wake cycle, while POA inhibition increased wakefulness without impacting sociability or social memory. These findings suggest that MS^GABA^ hyperactivity inhibits downstream regions, resulting in these behavioral deficits (Figure 1). Finally, the authors tested whether activating POA and CA2 neurons could rescue the behavioral deficits in *Nlg3*-cKO mice. Activation of CA2 neurons innervated by MS^GABA^ terminals restored social memory, increasing interactions with a novel mouse. Activation of POA neurons innervated by MS^GABA^ terminals increased NREM sleep and decreased wakefulness.

Together, this study reveals a role for MS^GABA^ neurons in regulating social deficits and sleep disturbances through distinct downstream projections. While these findings provide insights about MS^GABA^ hyperactivity in autism, it raises questions about the broader implications and potential applications of this research.

## Unanswered questions

Sun et al. (16) discovered social memory and sleep impairments following *Nlg3* cKO in the MS, uncovering a neural circuit change underlying autism. Still, unanswered questions remain. The optrode recordings revealed that MS^GABA^ neurons of *Nlg3*-cKO mice, but not control mice, encoded social approach and avoidance. A key question is how *Nlg3*-cKO MS^GABA^ neurons acquired these encoding properties underlying social behavior.

As NLG3 is a critical postsynaptic molecule involved in synapse organization, MS *Nlg3* deletion possibly alters synaptic properties of MS^GABA^ neurons. This effect is likely not limited to GABAergic neurons, as NLG3 is found at excitatory and inhibitory synapses (17). Such changes could influence multiple synapses, impacting properties like synaptic plasticity. For example, previous studies have shown a loss of cerebellar long-term depression in *Nlg3*-knockout mice (18) and enhanced hippocampal long-term potentiation in *Nlg3*-mutant mice (19).

Perhaps NLG3-driven changes in synaptic plasticity affect the encoding capabilities of MS^GABA^ neurons. A recent study has suggested that hippocampal long-term potentiation enables neurons to encode reward and novelty (20). This finding could explain why *Nlg3*-CKO mice, but not controls, encoded approach and avoidance. However, it remains unclear why changes to MS synapses in particular affect behavior. The MS mediates various arousal-based behaviors (21–23), so perhaps this region monitors overall internal state changes. Additional studies should investigate how *Nlg3* cKO affects MS synaptic plasticity and approach and avoidance computations.

## Future directions

These discoveries open several avenues for future research. Sun et al. (16) link social memory and sleep impairments to *Nlg3* cKO in the MS. However, the mechanism by which *Nlg3* cKO induces MS^GABA^ hyperactivity remains an open question. Previous studies have indicated that NLG3 knockdown reduces excitatory synaptic strength (24), increases inhibitory and decreases excitatory miniature events (19), and disrupts tonic endocannabinoid signaling, enhancing GABA release probability (25). While these changes may contribute to MS^GABA^ hyperactivity, further research should further probe the involved mechanisms. Similarly, whether these observed effects vary by sex, persist throughout the lifespan, or are modulated by hormonal or genetic factors remain to be studied. Ultimately, the Sun et al. findings open doors for exploring targeted pharmaceutical interventions that could mitigate deficits in social memory and sleep in ASD.

## Version 1 10/01/2024

Electronic publication
